# Analysing Powder Injection Moulding of a Helix Geometry Using Soft Tooling

**DOI:** 10.3390/polym13234183

**Published:** 2021-11-29

**Authors:** Alberto Basso, Yang Zhang, Jacob Kjeldahl Pløger, Jon Spangenberg, Hans Nørgaard Hansen

**Affiliations:** Department of Mechanical Engineering, Technical University of Denmark, 2800 Copenhagen, Denmark; yazh@mek.dtu.dk (Y.Z.); s145012@student.dtu.dk (J.K.P.); josp@mek.dtu.dk (J.S.); hnha@mek.dtu.dk (H.N.H.)

**Keywords:** powder injection moulding, additive manufacturing, soft tooling, low pressure injection moulding, simulation, freeform injection moulding

## Abstract

Freeform injection moulding is a novel technology for powder injection moulding where a sacrificial 3D printed mould (i.e., a soft tooling) is used as an insert in the injection process. The use of 3D printed moulds enable a higher geometrical design flexibility as compared to the conventional injection moulding process. However, there is still very limited knowledge on how the sacrificial soft tooling material and powder suspension handles the increased geometrical complexity during the process. In this study, a stainless steel powder suspension is injected into a geometrically challenging sacrificial mould (viz. a helix structure) that is produced by vat photopolymerization additive manufacturing. Computed tomography is used to quantify the geometrical precision of the mould both before and after injection. In addition, a new numerical model that considers the suspension feedstock is developed to investigate the powder injection moulding process. The numerical results are found to be in qualitative good agreement with the experimental findings in terms of pinpointing critical areas of the structure, thereby highlighting a new pathway for evaluating sacrificial inserts for powder injection moulding with a high geometrical complexity.

## 1. Introduction

Powder Injection Moulding (PIM) is a well-established technology for mass manufacturing of metal parts. Nowadays the market demands new and highly customized products, forcing the producers to continuously update the moulds in their manufacturing processes. Applications of PIM include dental implants [1,2], artificial joints [3,4], medical hand tools for special surgical operations [5,6] and steel cutting tools [7,8]. PIM is a profitable process for mass production, but in the case of prototyping or small batch production, the cost of the metal tools has an influential weight on the overall cost of the final parts [9]. An efficient way to reduce the manufacturing cost of PIM parts, when only a low number of units need to be produced, is to integrate Additive Manufacturing (AM) with PIM. In addition to being more affordable, the use of AM in a PIM process chain (also called soft tooling) opens new avenues for the possible geometry of the final part, which overcomes some limitation of the traditional PIM process [10]. Moulds for soft tooling in PIM are generally produced via Material Extrusion Additive Manufacturing [11] or Vat-Photopolymerization Additive Manufacturing (VPAM) [12,13]. In the case of VPAM, it is possible to manufacture sacrificial moulds with special resins that can be dissolved chemically [14] or thermally [15]. This soft tooling technique is also known as freeform injection moulding or lost form injection moulding. The advantage of freeform injection moulding is its ability to produce complex geometries like a helix structure; however, there is a lack of knowledge for powder injection moulding with such complex structures, since they are not possible to produce with conventional injection moulding. One of the challenges of using a sacrificial mould, or a soft tool in general, is that the strength of this material is lower than that of traditional tool steel [16], which hinders the possibility to use injection pressures from 60 to 200 MPa that are typically applied in conventional powder injection moulding.

Numerical models have been extensively used to analyse various polymer processing technologies [17], such as fused deposition modelling [18,19,20] and resin injection pultrusion [21,22]. In case of injection moulding, simulations have also been successfully used in both research and industry [23,24]. A good overview of simulations of the powder injection moulding process commonly used in industry can be found in the work of Yavari et al. [25] and Raymond [26], but only limited work exists that looks into simulations of low pressure powder injection moulding [27,28,29]. A knowledge gap is found when dealing with simulations of low pressure powder injection moulding with a high geometrical complexity.

The aim of this work is for the first time to analyse the fabrication of a geometrically complex helix structure when using low pressure injection moulding in combination with a sacrificial mould that is produced by VPAM. Computed tomography is used to determine the geometry of the soft tool before injection as well as the displacements in the part over the course of the process. In addition, a novel numerical process model is developed to simulate the flow and packing behaviour of the stainless steel powder suspension during the PIM process. The experimental and numerical results are used to understand the deformation pattern in the helix structure.

## 2. Materials and Methods

### 2.1. The Freeform Injection Moulding Process Chain

**Figure 1:**
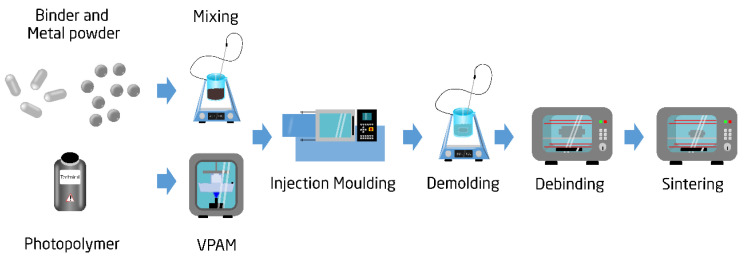
The freeform injection moulding process chain.

In Figure 1, a process chain for PIM is illustrated. A detailed description of this process chain can be found in the authors’ previous works [16]. In this study, the tools used for the injection moulding machine were fabricated using VPAM. The photopolymer resin applied in the VPAM process was water soluble, which enabled the creation of the helix structure. The feedstock for the low pressure injection moulding was tailor made by mixing together 316L stainless steel powder (with an average size of 50 µm) with a binder system based on low density polyethylene and paraffin wax. This feedstock was selected due to its low viscosity (below 50 Pa s at 120 °C), as it reduced the pressure during injection. The characterization of the feedstock can be found in a previous investigation [30]. The solid loading of the feedstock was 70% in volume.

The fabricated moulds were injected using a BOY XS 10 ton injection moulding machine from Dr. Boy, Neustadt-Fernthal, Germany. Table 1 shows the injection moulding conditions used during the experiment. To avoid mould distortion, the injection pressure was kept below 15 MPa. After injection, the sacrificial mould was dissolved in an alkaline solution, and the green body was obtained. Note that in this investigation, the deformation in the helix structure was evaluated after the demoulding step, and thus the debinding and sintering process steps were not considered.

### 2.2. Investigated Geometry

**Figure 2:**
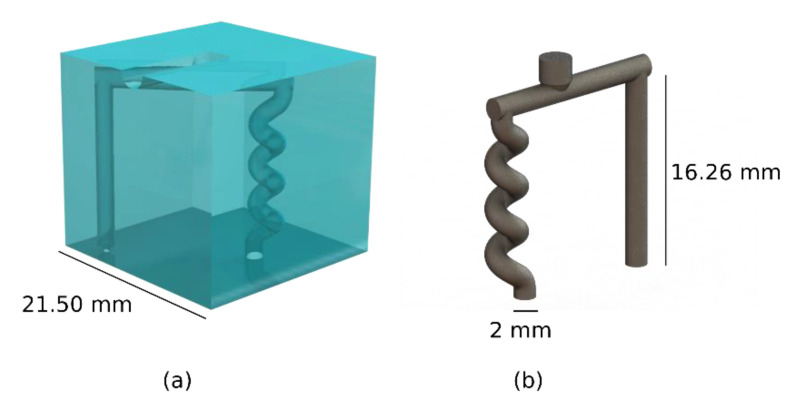
Three-dimensional printed sacrificial mould (**a**) and rendering of the injected part after mould dissolution (**b**).

A mould with a straight pillar and helix structure was used (see Figure 2). A total of 5 moulds were produced and injected. The straight pillar was designed with a height of 16.26 mm and diameter of 2 mm. Such simple geometry was selected to easily detect distortion and deflection of the cavity after the injection. The helix structure had the same overall height and diameter of the pillar, with three revolutions and a pitch of 4 mm. The geometrical deviation of the part was measured using a Werth Messtechnik Tomoscope XS CT scan from Werth Messtechnik, Gießen, Germany, with a pixel size of 51 µm. Specifically, the deviation between the point cloud obtained by the CT scan and the CAD file was measured using the software GOM inspect.

### 2.3. Numerical Model

**Figure 3:**
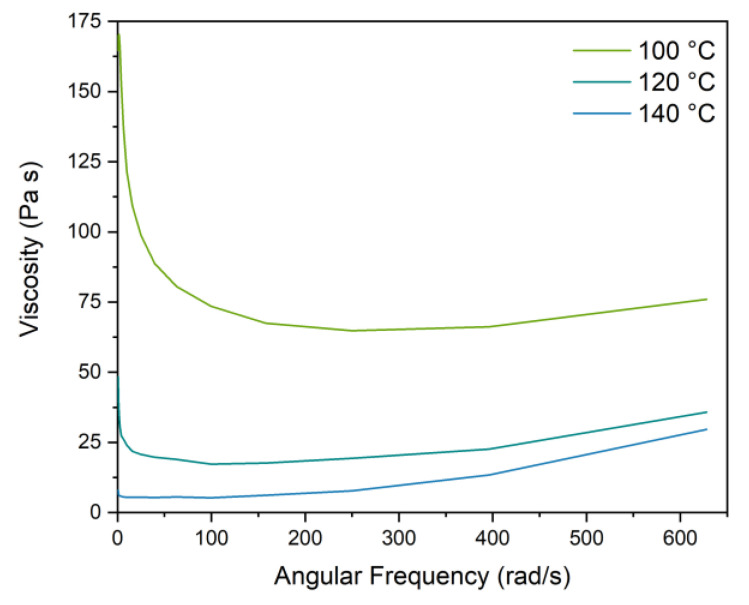
Viscosity vs. angular frequency measured at 100 °C, 120 °C and 140 °C.

The numerical PIM model was developed in Autodesk Moldflow synergy 2019 (AMS), jointly with Autodesk Moldflow Data Fitting (AMDF). The numerical model simulates the injection and packing step of injection process, but not the solidification phase. The numerical model considered four governing equations: conservation of mass, conservation of momentum, energy balance and particle-phase mass balanced [31], listed hereinafter. The conservation of mass equation is as follows:

(1) $$\frac{\partial \rho }{\partial t}+\nabla \cdot (\rho \;\vec{V})=0$$

where ρ is the density of the suspension, *t* represents the time and $\vec{V}$ is the velocity vector. The conservation of momentum equation is as follows:

(2) $$\rho \frac{D\vec{V}}{Dt}=-\nabla P+\nabla \cdot \;\overset{⇉}{\tau }+\rho \vec{g}$$

where *P* is the pressure, $\overset{⇉}{\tau }$ is the viscous stress tensor and $\vec{g}$ is the gravitational acceleration vector. The conservation of energy equation is as follows:

(3) $$\rho C_{p}\frac{DT}{Dt}=\nabla \cdot (k\nabla T)+\overset{⇉}{\tau }:\nabla \vec{V}+\beta T\frac{DP}{Dt}$$

where $C_{P}$ is the specific heat capacity, T is the temperature, k is the thermal conductivity and β is the expansivity. The latter is a function of the variation of the density of the suspension with the temperature. Finally, the particle-phase mass balance equation is given by

(4) $$\frac{\partial \theta }{\partial t}+\vec{V}\cdot \nabla \theta =-\nabla \cdot {\vec{J}}_{⏊}$$

where θ is the volume fraction of powder and ${\vec{J}}_{⏊}$ is the particle migration flux, which depends on the viscosity of the suspension, the dimension of the particles and particle sedimentation. AMDF was used to create the feedstock (i.e., metal powder suspension) for the simulation. The viscosity data of the feedstock was measured at different temperatures (see Figure 3) and imported in the software using the WLF viscosity model [32]. This model correlates the variation of the viscosity of the feedstock with the shear rates:

(5) $$\eta \left(\dot{\gamma }\right)=\frac{\eta_{0}}{1+{\left(\frac{\eta_{0}\dot{\gamma }}{\tau^{*}}\right)}^{1-n}}$$

where $\eta_{0}$ is the viscosity at zero shear rate, $\dot{\gamma }$ represents the shear rate, n is the power law index and $\tau^{*}$ is the critical shear stress at the shear thinning transition. The viscosity at zero shear stress is given by

(6) $$\eta_{0}=D_{1}\times \exp\left[\frac{A_{1}\left(T-D_{2}\right)}{A_{2}+T-D_{2}}\right]$$

where A_1_, A_2_, D_1_ and D_2_ are coefficients that are fitted to the viscosity curves in Figure 3. A detailed investigation on the viscosity of the feedstock can be found in the authors’ previous work [30]. 

The numerical model solved the governing equations with a 3D coupled algorithm [33]. The mesh type was 3D tetrahedral elements with a total number of ~135,000. In addition, the model used the level-set method to track the free surface of the feedstock [34,35]. The thermal and mechanical properties of the feedstock were acquired from the work of Marhöfer et al. [36] and Lui et al. [37]. The properties are summarized in Table 2.

## 3. Results and Discussion

**Figure 4:**
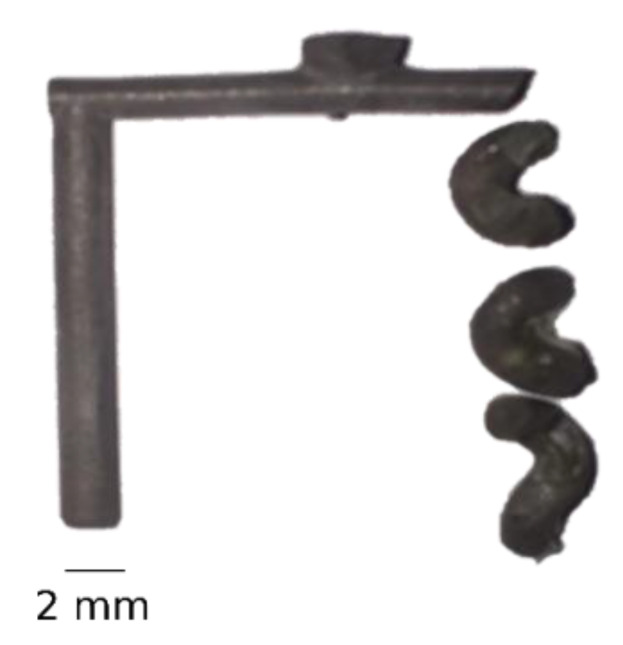
Part after demoulding. All samples showed an intact pillar, while the helix broke at the turning points.

After injection and dissolution of the moulds, all the parts showed damage at the helix structure (see Figure 4). The parts broke at the turning point of the helix structure, while the straight pillars stayed undamaged. The failure of the spiral is analysed in the next subsections using CT and numerical modelling.

### 3.1. Geometrical Displacement 

**Figure 5:**
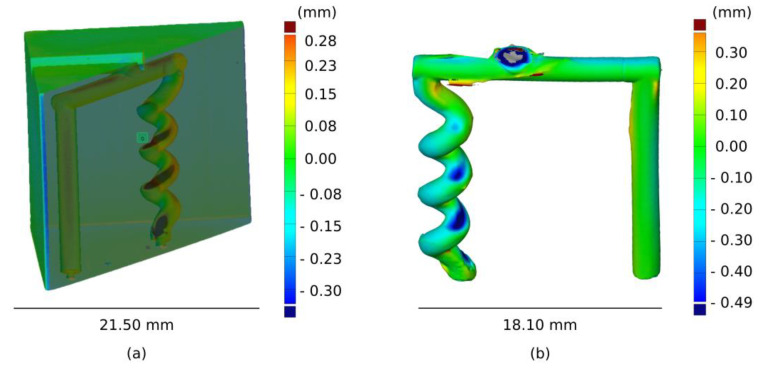
CT scan of an empty cavity (**a**) and filled cavity (**b**). Colour scale illustrates the geometrical displacement from the CAD file.

A CT scan was used to investigate the dimension of the cavity of the mould. Figure 5a shows the displacement for the empty mould. While the cavity of the straight pillar was printed with high fidelity, deviations were detected at the turning points of the helix. The displacement between the nominal design and scan was larger than 0.3 mm, showing a size decrease in the printed cavity. The necking in the helix channel was caused by an incomplete cleaning. After printing, the cavity of the mould was filled with uncured resin, which needed to be removed with isopropyl alcohol and compressed air. In case of thin intricate channels, such as helix structures, the complete removal of uncured resin can be challenging. If some leftover resin is still present after cleaning, it will harden during the post curing process, generating the observed size decrease at the turning points. The variation of the cavity dimension could also be seen directly in the filled part (see Figure 5b). The helix structure deviated from the CAD file at the turning points, and the deviations seemed to be more severe towards the bottom. This indicates that the uncured resin during the cleaning process tends to accumulate towards the end of the structure. At the end of the helix, there was only one venting hole (0.8 mm) from which the uncured resin could escape; such a narrow feature might hinder the complete cleaning of the cavity. The deformation of the helix created weak points that could be the source of failure during demoulding. 

### 3.2. Shear Induced Defects 

**Figure 6:**
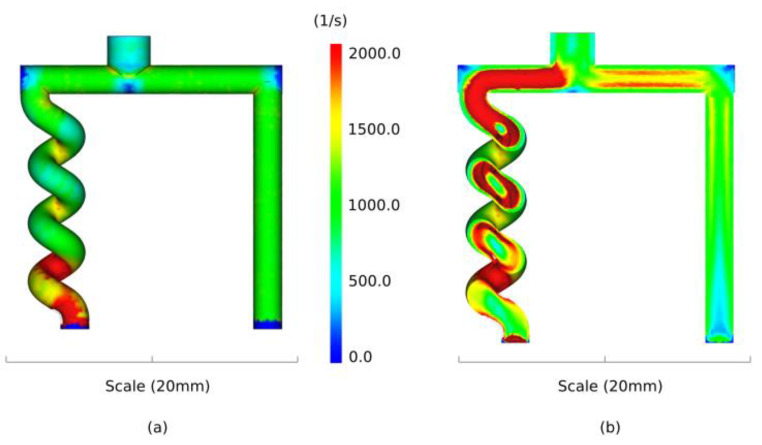
Simulation of the shear rate in the cavity (**a**) and cross section of the cavity (**b**).

In Figure 6, the shear rates predicted by the developed numerical model are shown. The shear rates are depicted at the end of the injection. The numerical results illustrate that the highest variations in shear rates are obtained in the helix structure with values up to 2000 1/s. Since the feedstock is shear sensitive, the fluctuation in shear can cause powder binder separation, as reported in [38].

**Figure 7:**
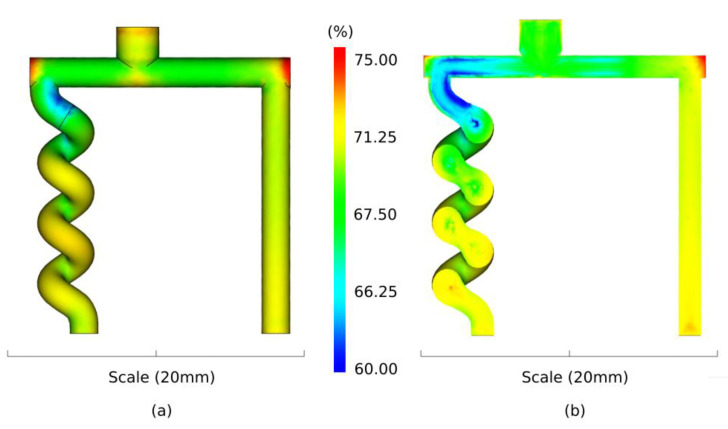
Simulation of the powder volume concentration for the entire cavity (**a**) and for the cross section (**b**).

Figure 7 shows numerical predictions of the powder volume concentration for the entire cavity and for its cross section. The powder volume concentration is approximately 70% in the pillar (i.e., homogeneous), while variations from 60% to 73% are detected in the helix structure with an increase in powder content from top to bottom. Variations in shear rate and powder volume concentration have also been shown by authors studying conventional powder injection moulding [39,40]. In particular, Tseng et al. [40] showed higher segregation at the turning point of an L-shaped structure due to variations in shear rate. The segregation pattern in the helix structure might be the reason for the helix structure’s inability to withstand the dissolution process, which would be in accordance with other researchers’ findings [41,42,43,44].

### 3.3. Pressure Difference in the Cavity 

**Figure 8:**
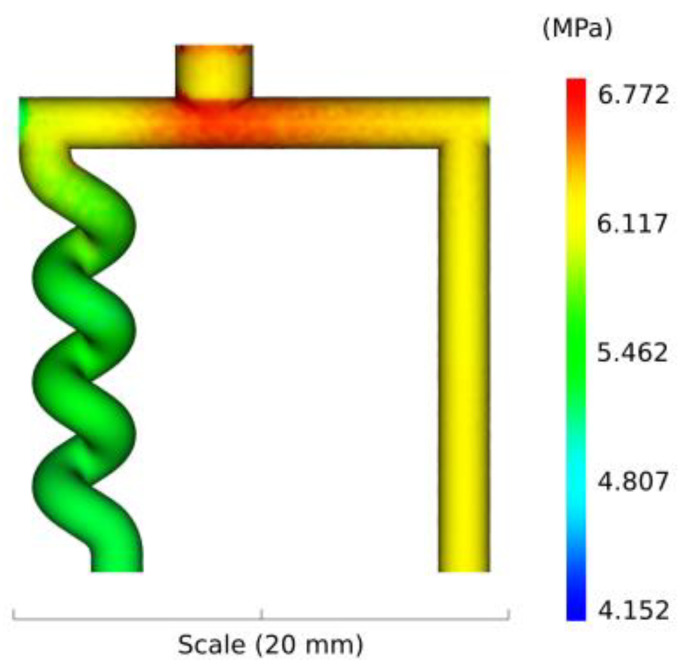
Numerical results of the pressure at the end of the injection.

Figure 8 presents the numerical results of the pressure at the end of the injection. A lower pressure is obtained in the helix structure as compared to the pillar. However, the difference in pressure between the two cavities is quite low: 5.5 MPa in the helix and 6.1 MPa in the pillar. The small variation in pressure indicates that this probably is not the origin of the failure of the helix. 

### 3.4. Shrinkage of the Part 

**Figure 9:**
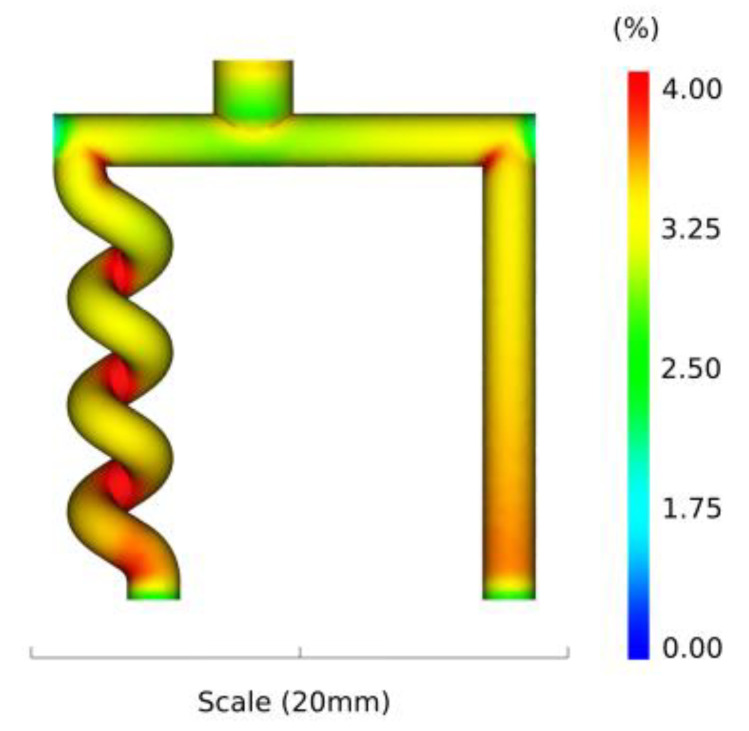
Simulation of the volumetric shrinkage of the part.

In Figure 9, the numerical results of the volumetric shrinkage of the part are plotted at the end of the packing step of the injection moulding process. The simulation shows the highest shrinkage as well as the variation in shrinkage in the helix structure. Up to 4% variation in shrinkage was detected at the turning point of the helix. This shrinkage profile might generate internal stresses and crack formation that potentially can lead to failure of the structure. Hsu et al. [45] observed this exact pattern when studying conventional powder injection moulding, both experimentally and numerically. 

## 4. Conclusions

This paper investigated the behaviour of a powder suspension when injected into a 3D printed sacrificial mould with high geometrical complexity. The soft tool had a straight pillar and a helix structure both with a diameter of 2 mm. After demoulding of the parts, damage was observed at the helix. Specifically, the helix was broken at the turning points, illustrating the inability of the process to produce such a structure with a diameter of 2 mm. A CT scan and numerical model were used to analyse the failure of the parts. The CT scan of the empty mould revealed the presence of necking at the turning points of the helix with a deviation of more than 300 µm from the nominal design. These variations in the cavity dimension were also confirmed by the CT scan of the part. The geometrical alteration of the helix structure created weak spots that could act as the source of failure. In addition, the numerical model predicted shear rates up to 2000 1/s, powder volume concentrations from 60% to 73% and volumetric shrinkage up to 4%, which all could be alternative sources of the failure of the helix. From a qualitative point of view, the numerical results were congruent with the experimental findings in terms of highlighting the critical areas of the helix structure. In future research, a quantitative comparison between the experimental and numerical results should be carried out. In addition, it is of interest to find the appropriate diameter of the helix structure and process parameters that does not lead to failure.

## Figures and Tables

**Table 1:** Injection moulding conditions.

Properties	Value
Injection temperature at nozzle	120 °C
Injection pressure	15 MPa
Packing pressure	12 MPa
Packing time Mould temperature	3 s 20 °C

**Table 2:** Properties used in the simulation for the feedstock.

Properties	Value	Source
**Viscosity Model** **WLF coefficients**		Fitted data from rheology measurements
N	0.5699	
Taus	4.93 × 10^4^ Pa	
D_1_	1.84 × 10^16^ Pa s	
D_2_	263.15 K	
A_1_	47.62	
A_2T_	51.60 K	
**Thermal Conductivity**		[36]
At 298 °C	1.95 W/mK	
At 383 °C	1.40 W/mK	
At 453 °C	1.59 W/mK	
**Specific Heat**		[36]
At 298 °C	640 J/kgK	
At 383 °C	900 J/kgK	
At 453 °C	710 J/kgK	
**Mechanical properties**		[37]
Young’s Modulus	0.82 GPa	
Poisson’s ratio	0.40	
In plane shear modulus	0.29 GPa	
**Thermal Properties**		[37]
Coefficient of linear thermal expansion	83.93 × 10^−6^/°C	
Softening point	71.90 °C	
Transition temperature	53.92 °C	

## Data Availability

The data presented in this study are available on request from the corresponding author.
